# Supplementary material for: β-aminopropionitrile-induced thoracic aortopathy is refractory to cilostazol and sildenafil in mice
Source: PLoS One. 2025 Aug 29;20(8):e0322434. doi: 10.1371/journal.pone.0322434 (PMC12396674; doi:10.1371/journal.pone.0322434)
Supplement: Attachment — Submitted filename: Response to Reviewers.docx [file pone.0322434.s003.docx]

**Response to Reviewers’ Comments:**

**Manuscript ID:** PONE-D-25-15324

**Title:** β-aminopropionitrile-induced thoracic aortopathy is refractory to cilostazol and sildenafil in mice

We appreciate the constructive comments of the reviewers that have assisted us in improving this manuscript. We have provided point-by-point responses to the critiques and edited the manuscript accordingly. Reviewers’ comments are in *italic*, followed by our responses. Changes in the revised manuscript are represented in blue font. We hope the Editor and Reviewers agree that this revision has addressed the reviewers’ concerns.

**Reviewer #1**

1. *Novelty: Some work has been done with β-aminopropionitrile in rodents. A PubMed search with the term “β-aminopropionitrile aorta mice” yielded ~130 papers. Indeed, some of this work is from the authors. For example, Arterioscler Thromb Vasc Biol. 2024 Jul;44(7):1555-1569. Of note this latter paper provides details about the murine model. But this raises the question, why the treatment was not applied to mice when they were phenotype analysis for the 2024 paper. While data in both papers seems helpful, I do not understand why the authors did not combine the work into a single paper.*

Authors’ reply:

The previous study was designed to evaluate regional differences in BAPN-induced aortopathies between the ascending and descending aortas, while the present study focuses on the effects of PDE3 and PDE5 inhibition. Due to their distinct objectives, we did not combine the results of these two studies.

2. *Rationale: The Introduction offers a rationale based on data showing conflicting effects of the two treatments with one limiting aneurysm formation and the other worsening them. Thus, it seems reasonable to consider what accounts for this. However, in truth the models of arterial disease were not the same so that alone might be a reason for using one model and testing both agents.*

Authors’ reply:

As the reviewer pointed out, previous studies evaluated the effects of cilostazol and sildenafil on abdominal aortic aneurysms using several mouse models. However, their effects in thoracic aortopathy remain unknown. Therefore, we examined both drugs in a thoracic aortopathy mouse model of BAPN administration in C57BL/6J mice

3. *Methods: The text on the timing of treatments and injury is a little confusing. Also, why give β-aminopropionitrile for 28 or 84 days? What was the reason for looking at plasma levels of the treatments; perhaps as a means to estimate the effective delivered amounts? Did you suspect that mice were not taking the agents by eating less. How many samples over time were taken and how did this compare to the administration of the treatments. I would be surprised that half-lives of these agents are not published for mice and rats. Provide any available data on food and water intake, activity, and weight changes during the study intervals. Justify using just male mice. Provide a power analysis for cohort sizes. Provide any vitals and cardiovascular information on the mice -BP, pulse, cardiac output, ejection fraction, etc. if available. If not give us reasons why this was not checked as a method to ascertain if the agents made any difference globally, and not just at the aorta.
   I am concerned about the study design. As the authors published already on the high mortality of the stress (>40%) why go out to the extent that the same was achieved in the present study. This would seem to be unnecessary as one could look at early time points to find if the treatments made any difference.*

Authors’ reply:

We appreciate the reviewer’s detailed comments regarding the study design and methodology. Please find our point-by-point response below:

1. **Timing of Treatment and BAPN Injury:** In this study, BAPN was administered for two intervals, 4 and 12 weeks, to evaluate the effects of cilostazol and sildenafil on both the initiation (4 weeks) and progression (12 weeks) of thoracic aortopathy.
2. **Determination of Drug Delivery:** To validate drug delivery, we measured plasma concentrations of the administered drugs at the end of the experiment. While we did not directly monitor food or water intake, we continuously tracked body weights throughout the study. No significant differences in body weight were observed between groups (**Figure for the Editor and Reviewers**), indicating no difference in food consumption that would imply consistent drug administration. There was comparable overall health status among the groups.

**Figure for the Editor and Reviewers.** Body weight was measured weekly during BAPN administration by 3-week (left) and 9-week (right) of BAPN administration.

3. **Half-life of cilostazol and sildenafil:** Although the plasma half-lives of cilostazol and sildenafil in mice have not been reported, their half-lives in rats have been reported, as cited in references (21) and (22).
4. **Use of Male Mice:** We acknowledge the importance of sex as a biological variable. Our previous study (PMID: 38779856) showed that BAPN-induced thoracic aortopathy does not exhibit significant sexual dimorphism, suggesting that the effects of cilostazol and sildenafil would not differ markedly between sexes. Therefore, given the lack of sexual dimorphism in this model, we focused on male mice in this study following the “3Rs” (Replacement, Reduction, and Refinement) of ARRIVE guidelines.
5. **Power Analysis and Sample Sizes:**
   Based on our previous study (PMID: 38779856), we designed the present study with a sample size of n = 15 per group. This was calculated to achieve a statistical power greater than 0.8 to detect a 1.1 mm difference with a standard deviation of 0.9, assuming an alpha level of 0.05. Since this sample size was sufficient to yield robust conclusions, no additional studies were performed.
6. **Cardiovascular Parameters:**
   As our study was designed to focus specifically on thoracic aortopathy rather than cardiomyopathy, we did not collect cardiac or hemodynamic data.
7. **Study Duration and Mortality:**
   As noted by the reviewer, the BAPN model exhibits high mortality due to aortic rupture and dissection, approximately 60% by 4 weeks and 80% by 12 weeks. In addition to aortic rupture and dissection, BAPN induces thoracic aortic aneurysm formation, which requires 4 to 12 weeks of continuous administration. Based on our hypothesis that cilostazol protects, while sildenafil exacerbates BAPN-induced thoracic aortopathy, we selected these intervals to evaluate phenotypic differences among groups.

We hope these clarifications address the reviewer’s concerns.

4. *Data: The whole tissue mRNA data is not too helpful. What should be done is single cell seq followed if indicated with bulk analysis. We just do not what to make of increase PDE mRNA - is it form immune cells, fibroblasts, smooth muscle cells, endothelial cells. They all respond to NO and cGMP and so have counter controls like PDEs. I suggest trying to localize this information. Maybe high contrast and magnification immunofluorescence of the aortic walls would help. How did you validate the morphometric approaches used on the resected arch and descending aorta? I think in vivo doppler or other non-invasive approaches would be much more helpful here. The paper has a limit in mechanism. To begin why not map the NO-cGMP-PKG pathway in the samples from the mice; the target might be PKG. As well, while transcripts to several PDEs changed there is no explanation as to why? Was it feedback from a lock of NO-cGMP signal. And what would account for the loss of NO-cGMP or any relative resistance to the same? Did the β-aminopropionitrile cause secondary/tertiary modifications on pathway molecules that altered their function? And/or did it upregulate known inhibitors of the NO-cGMP-PKG cascade such as thrombopsindin-1, superoxide, etc. (see: Proc Natl Acad Sci U S A. 2005 Sep 13;102(37):13141-6 and Nat Rev Cancer. 2009 Mar;9(3):182-94). I would bet a link to β-aminopropionitrile and superoxide is hinted at but not fully defined, while low NO turns on thrombospondin-1 expression (see Proc Natl Acad Sci U S A. 2005 Sep 13;102(37):13147-52).*

Authors’ reply:

We appreciate the reviewer’s insightful comments. We fully agree that mechanistic studies, such as single-cell RNA sequencing, spatial localization of PDE expression, and evaluation of the NO–cGMP–PKG signaling axis, would be highly valuable. As the reviewer notes, several cell types, including smooth muscle cells, fibroblasts, immune cells, and endothelial cells, respond to NO and cGMP, and a better understanding of the cell-specific regulation of PDEs is indeed important. However, we prioritized reporting the phenotypes that neither cilostazol nor sildenafil influenced BAPN-induced thoracic aortopathy as the main message of the study. We believe it is important to share these findings promptly, as they suggest that PDE-mediating therapies may not be broadly effective in all models of thoracic aortopathy.	Regarding morphometric validation, we used standardized measurements from resected aortic segments and ensured consistency across samples. We agree that in vivo non-invasive imaging would be informative and aim to incorporate such approaches in future investigations.

5. *Legends: Add the exact statistics test used for each graph.*

Authors’ reply:

Statistical tests used have been described in each figure legend.

6. *Figures: Ok but was all of the color needed?*

Authors’ reply:

We hope the editor and reviewers agree that the color code helps readers to understand the study design easily.

7. *Indicate if AI was or was not used in manuscript perpetration and a section on what each author contributed. As well, mention somewhere that the paper is online as a pre-print.*

Authors’ reply:

The manuscript has been revised to include a description of each author's contributions. Grammarly and the built-in spell-check function of Microsoft Word were used for grammar and spelling checks. Although the manuscript has been uploaded to the bioRxiv preprint server, we have not mentioned this within the manuscript itself, in accordance with the manuscript preparation guidelines for PLoS ONE.

**Changes in the Manuscript**

Line 336:

**Authors’ Contributions**

SCT and HS analyzed the data, prepared figures, and wrote the main manuscript text. SCT, SI, JCH, MKF, and DAH implemented animal experiments. SCT, HSL, and AD contributed to the study design. All authors reviewed the manuscript.

Line 344:

Grammarly and the built-in spell-check function of Microsoft Word were used for grammar and spelling checks.

**Reviewer #2**

1. *Inclusion and Exclusion Criteria: Not detected. Please specify any pre-defined criteria used to determine whether participants, animals or samples were eligible for the study, or exclude participants, animals or samples from the study.*

Authors’ reply: We have revised the manuscript to describe that no mice were excluded from the study.

**Change in the Manuscript**

Line 92: Mice were randomly assigned to the study groups, and no mice were excluded from the study.

2. *Flow charts and attrition: We did not find a study flow chart of excluded observations. We strongly recommend using flow charts because they provide an overview of the study design and more information about attrition. If you included a study flow chart in your supplemental files, we apologize for missing this. Our tool is not able to screen separate supplemental files. Sentence about attrition: not detected. Please provide information about the drop-out of subjects, or loss of animals or samples. This could be done using a study flow chart, or described in the text.*

Authors’ reply: As no mice or samples were excluded during the study, there were no cases of attrition to report. We hope that the Editor agrees that a study flow chart is not necessary in our manuscript.

3. *Subject Demographics: Age: not detected. Weight: not detected.*

Authors’ reply: The age and weight have been added to the revised manuscript.

4. *Randomization: Not detected. If you performed an experimental study, please specify whether participants, animals or samples were randomly assigned to treatments or groups, and specify the randomization procedure.*

Authors’ reply: We have revised the manuscript to clarify that the study mice were randomly assigned to the experimental groups.

5. *Power Analysis: Not detected. Please state how you determined the sample size for the study. If you performed a power or sample size calculation, please provide details.*

Authors’ reply: Based on our previous study (PMID: 38779856), we designed the present study with a sample size of n = 15 per group. This was calculated to achieve a statistical power greater than 0.8 to detect a 1.1 mm difference with a standard deviation of 0.9, assuming an alpha level of 0.05.

**Change in the Manuscript**

Line 89: Based on our previous study (6), the present study was designed with a sample size of n = 15 per group to achieve a statistical power greater than 0.8 to detect a 1.1 mm difference with a standard deviation of 0.9, assuming an alpha level of 0.05.

6. *C57BL/6J suggested: RRID: IMSR_JAX:000664. Please make sure this RRID suggestion is accurate before including in your manuscript.*

Authors’ reply: We have confirmed the RRID and included it in the manuscript."

7. *Millipore-Sigma suggested: None. Please add an RRID to help others identify this resource.*

Authors’ reply: Millipore-Sigma is not in the RRID database.

8. Genome Expression Omnibus suggested: None. Please add an RRID *to help others identify this resource.*

Authors’ reply: The RRID for Genome Expression Omnibus has been added to the manuscript.

9. *edgeR suggested: None. Please add an RRID to help others identify this resource.*

Authors’ reply: The RRID for edgeR has been added to the manuscript.

10. *SigmaPlot suggested: None. Please add an RRID to help others identify this resource.*

Authors’ reply: The RRID for SigmaPlot has been added to the manuscript.

11. *Open code: If you wrote code to analyze your data, please consider sharing the code in a public repository. This makes it easier for others to reproduce your analyses, and may also aid others seeking to analyze similar datasets.*

Authors’ reply: We will make the R code used in this manuscript available upon request.

12. *Open data: If permitted, sharing data on a public repository with a persistent identifier (a DOI or accession number) can improve reproducibility and make it easier for other scientists to expand on your work. Papers with open data are cited more often than papers without open data. Some institutions have an expert who can provide advice on data sharing.*

Authors’ reply: We have shared all data as a supplemental data file.

13. *Self-acknowledged limitations: An explicit section about the limitations of this study was not found. We encourage authors to include a paragraph in the discussion that addresses study limitations. Every study has limitations. Describing these limitations helps readers to understand and contextualize the research.*

Authors’ reply: We would like to note that the limitations of our study have been discussed in the third, fourth, and fifth paragraphs of the Discussion section in this revision of the manuscript.
