# Supplementary material for: β-aminopropionitrile-induced thoracic aortopathy is refractory to cilostazol and sildenafil in mice
Source: PLoS One. 2025 Aug 29;20(8):e0322434. doi: 10.1371/journal.pone.0322434 (PMC12396674; doi:10.1371/journal.pone.0322434)
Supplement: Attachment — Submitted filename: Comments.docx [file pone.0322434.s004.docx]

Comments:

The author did the research on two inhibitor drugs (phosphodiesterase inhibitors), one is cilostazol a PDE3 inhibitor and other is sildenafil a PDE5 inhibitor, to investigate the effect of these two drugs on the development and progression of disease thoracic aortopathy induced by β-aminopropionitrile (BAPN) in mice. Author also did bulk RNA sequencing to see the β-aminopropionitrile (BAPN) effect on Pde3a and Pde5a gene transcription level. Although the author found both genes were upregulated in the disease development but dietary administration of both drugs failed to alter the outcomes i.e. aneurysm formation, aortic rupture, and mortality. Finally author concluded based on the experimental data that cilostazol and sildenafil did not influence BAPN-induced thoracic aortopathy in mice.

The author has investigated a very important disease of the concerned. The study is well structured and addressed an important question. The most interested thing is the different effect of cilostazol and sildenafil on the disease. However, there are several major concerns must be addressed.

Major comments:

1) Author please explain the hypothesis of your study, is it only to find out the efficacy the inhibitors? As it is not clear with your experimental plan.

2) The author used a dose of 1 mg/day for each drug, how was this optimization done? Other studies often used 30-60 mg/day. The author should provide a dose optimization data as the dose author used for the study is considerably low and maybe that’s why lack of therapeutic effects observed. Pharmacokinetic data would be helpful.

3) Please justify why author chose 84 days of administration of treatments?

4) The BAPN concentration used in this study (0.5% wt/vol) which showed high mortality rates. There are previous study with low concentration (0.1-0.3%) as we know the BAPN effects are highly dose dependent. Author needs to justify dose choice based on their hypothesis or may be it is related to model also.

5) Why does the author only provide one time point mass spectrometry data? We know that the half-lives of cilostazol and sildenafil in plasma mice are relatively short (cilostazol: 2-4 hours, sildenafil: 0.3-2 hours).

6) Author did mass spectrometry which confirm the drug presence in plasma but did not address wheather the concentration of drugs were maintained throughout the experimental period or not as the half-lives of both drugs are relatively short

7) The author did bulk RNA sequencing, I don’t know how it will be helpful to author rather single cell sequencing will be helpful but I can assume the author has large data set but there is no evidence (eg. Bubble plot) mechanistic insight of drug effects or any molecular readouts in downstream pathways and author did not mentioned which cell types are involved in this mechanism. So the study lacks mechanistic investigation beyond RNA- sequencing data.

8) Author did not included any data on PDE enzyme activity , cAMP/cGMP levels or any signaling related study which limits its focus why drugs were ineffective?

9) The author states in the discussion section "These results indicate that BAPN-induced thoracic aortopathy is mediated by mechanisms distinct from other aortopathy models" by reinforcing this point with evidence or by emphasizing the ECM-dominant and non-inflammatory BAPN pathology that limits efficacy.

10) This study lacks histological evaluation, my suggestion is to include immunostaining data which would deepen the mechanistic insights.

11) The author should explain the novelty of this study in relation to other published studies as the present condition of the study only reflects the characterization of two drugs efficacy in thoracic region otherwise author should provide mechanistictic explanation for their negative findings.

Minor Comments:

1)	In the abstract section line 29 author started with the describing about the disease but why “Phosphodiesterases (PDEs) regulate intracellular cyclic nucleotide concentrations” suddenly came please explain.

2)	In the Introduction section line 46 “A key pathological feature of thoracic aortopathy and changes” Please check the grammer.

3)	In line 53 author mentioned “This mouse model” which mouse model author wants to mention, please include that.
