# Supplementary material for: β-aminopropionitrile-induced thoracic aortopathy is refractory to cilostazol and sildenafil in mice
Source: PLoS One. 2025 Aug 29;20(8):e0322434. doi: 10.1371/journal.pone.0322434 (PMC12396674; doi:10.1371/journal.pone.0322434)
Supplement: Attachment — Submitted filename: Response_to_Reviewers_auresp_2.docx [file pone.0322434.s005.docx]

**Response to Reviewers’ Comments:**

**Manuscript ID:** PONE-D-25-15324R1

**Title:** β-aminopropionitrile-induced thoracic aortopathy is refractory to cilostazol and sildenafil in mice

We appreciate the constructive comments of the reviewer that have assisted us in improving this manuscript. We have provided point-by-point responses to the critiques and edited the manuscript accordingly. Reviewers’ comments are in *italic*, followed by our responses. Changes in the revised manuscript are represented in blue font. We hope the editor and reviewer agree that this revision has addressed the reviewer’s concerns.

**Reviewer #1**

1) *Author please explain the hypothesis of your study, is it only to find out the efficacy the inhibitors? As it is not clear with your experimental plan.*

**Authors’ reply:**

Thank you for the constructive suggestion. Based on preclinical and clinical evidence, we hypothesized that cilostazol (PDE3 inhibition) attenuates thoracic aortopathy, whereas sildenafil (PDE5 inhibition) exacerbates the disease. We have revised the introduction section to describe the hypothesis and better align it with our experimental design.

**Changes in the manuscript:**

Line: 72

Based on clinical and preclinical evidence, we hypothesized that cilostazol attenuates thoracic aortopathy, whereas sildenafil exacerbates it.

2) *The author used a dose of 1 mg/day for each drug, how was this optimization done? Other studies often used 30-60 mg/day. The author should provide a dose optimization data as the dose author used for the study is considerably low and maybe that’s why lack of therapeutic effects observed. Pharmacokinetic data would be helpful.*

**Authors’ reply:**

We apologize for the unclear description. This study was designed initially to examine the effect of sildenafil on thoracic aortopathy. To determine the dose, we reviewed previous publications using mice and found that most studies used doses ranging from 0.8 to 2 mg/day, assuming a mean mouse body weight of 20 g. Therefore, we selected a dose of ~1.3 mg/day, which falls approximately in the middle of the reported range. For cilostazol, we chose to administer the same dose (1.3 mg/day) to allow for a consistent experimental design. While this dose is within the range commonly used in previous studies, it remains unknown whether higher doses could more effectively attenuate thoracic aortopathy. In the present study, these doses did not affect BAPN-induced thoracic aortopathy, but we acknowledge the reviewer’s suggestion that administering higher doses may impact disease formation and progression.

We also agree with the reviewer that pharmacokinetic data will provide important insights into understanding drug efficiency. However, in our study, drugs were administered through incorporation in the diet. Thus, it is difficult to determine the timing of drug intake. In addition, it is anticipated that thoracic aortopathy may influence drug metabolism, complicating the interpretation of pharmacokinetic profiles. Therefore, acquiring pharmacokinetic data will require careful optimization of study protocols, including controlled drug delivery systems and timing of sampling, which will take considerable time to develop. Given that both drugs are widely used in clinical practice, we hope the editor and reviewer will agree that we prioritize reporting the phenotypic outcomes promptly. We have revised the manuscript to include a discussion of these limitations and the need for future studies to test higher doses and assess in vivo pharmacokinetics of cilostazol and sildenafil in the context of thoracic aortopathy.

**Changes in the manuscript:**

Line: 313

While many of these studies using sildenafil employed doses around 1.3 mg/day, some studies investigating cilostazol administered higher doses of up to 60 mg/day. Therefore, it remains unknown whether higher doses can attenuate thoracic aortopathy.

3) *Please justify why author chose 84 days of administration of treatments?*

**Authors’ reply:**

In our previous study (PMID: 38779856), we investigated the effects of the duration of BAPN administration on thoracic aortopathy. We found that 28 days of BAPN administration induced acute aortic pathologies, such as aortic hemorrhage and dissection, while extending the administration to 84 days leads to progression of pathology with significant vascular remodeling. Therefore, in the present study, we administered BAPN for both 28 and 84 days to evaluate the effects of sildenafil and cilostazol on both the initiation and progression of thoracic aortopathy, respectively. We have revised the manuscript to clarify this rationale.

**Changes in the manuscript:**

Line: 88

Our previous study revealed that BAPN administration for 28 days induced acute aortic pathologies, and 84-day administration results in progressive vascular remodeling. Therefore, BAPN was administered for 28 and 84 days to evaluate the effects of drugs on both the initiation and progression of thoracic aortopathy, respectively.(6)

4) *The BAPN concentration used in this study (0.5% wt/vol) which showed high mortality rates. There are previous study with low concentration (0.1-0.3%) as we know the BAPN effects are highly dose dependent. Author needs to justify dose choice based on their hypothesis or may be it is related to model also.*

**Authors’ reply:**

We agree that the pathological effects of BAPN are highly dose-dependent. In our previous publication (PMID: 38779856), we compared BAPN at concentrations of 0.1%, 0.3%, and 0.5% (wt/vol) to assess their impact on severity of thoracic aortopathy. The incidence of death due to aortic rupture was 0%, 60%, and 80% for 0.1%, 0.3%, and 0.5%, respectively. Based on these results, we selected 0.5% BAPN for the present study to ensure a sufficiently high disease incidence that would allow us to robustly assess phenotypic outcomes. We have revised the Methods section to describe the justification.

**Changes in the manuscript:**

Line: 334

Based on our previous studies examining the dose-response of BAPN,[6], the present study used 0.5% BAPN to ensure a sufficiently high disease incidence that would allow robust assessment of phenotypic outcomes. We acknowledge that this high concentration leads to higher mortality, potentially interfering with data interpretation for the sildenafil group, since we hypothesized that inhibiting PDE5 augments thoracic aortopathy.

5) *Why does the author only provide one time point mass spectrometry data? We know that the half-lives of cilostazol and sildenafil in plasma mice are relatively short (cilostazol: 2-4 hours, sildenafil: 0.3-2 hours).*

**Authors’ reply:**

In the present study, blood samples for mass spectrometry were collected between 8 and 10 AM. Given that mice are nocturnal and consume food predominantly during the night, it is reasonable to assume that drug delivery occurred within a few hours prior to blood collection. Despite sampling at a single time point, we observed significantly elevated plasma concentrations of both cilostazol and sildenafil compared to their respective controls. This suggests that the administered doses were effective administered to mice, even when accounting for the short half-lives of these agents. Nevertheless, we agree that a more detailed pharmacokinetic analysis involving multiple time points is essential to fully characterize drug exposure. We have revised the manuscript to include this important limitation and to emphasize the need for future studies incorporating frequent plasma sampling to better define the pharmacokinetics of cilostazol and sildenafil.

**Changes in the manuscript:**

Line: 319

Also, frequent plasma sampling and assessment of downstream signaling pathways, such as cyclic adenosine or guanosine monophosphates, are important to validate effective cilostazol and sildenafil administrations.

6) *Author did mass spectrometry which confirm the drug presence in plasma but did not address whether the concentration of drugs were maintained throughout the experimental period or not as the half-lives of both drugs are relatively short.*

**Authors’ reply:**

We appreciate the reviewer’s comment. This point is closely related to the previous comment regarding pharmacokinetics and the limited number of time points used for mass spectrometry. As noted, we measured plasma concentrations of cilostazol and sildenafil at a single time point, and both were significantly elevated compared to their respective controls. While this confirmed drug administration, we acknowledge that the short half-lives of these drugs raise an important question regarding whether effective plasma concentrations were sustained throughout the experimental period. As discussed in our revised response to Comment 5, the use of diet for drug administration complicates the precise assessment of drug intake timing and fluctuations in drug concentrations. To fully address this issue, future studies will require detailed pharmacokinetic profiling with multiple time points to determine whether therapeutic concentrations are maintained over time. We have revised the manuscript to include this point in the discussion (Line 318).

7) *The author did bulk RNA sequencing, I don’t know how it will be helpful to author rather single cell sequencing will be helpful but I can assume the author has large data set but there is no evidence (eg. Bubble plot) mechanistic insight of drug effects or any molecular readouts in downstream pathways and author did not mentioned which cell types are involved in this mechanism. So the study lacks mechanistic investigation beyond RNA- sequencing data.*

**Authors’ reply:**

We appreciate the Reviewer’s comment regarding the depth of mechanistic investigation. This study was designed to evaluate the effects of cilostazol and sildenafil on BAPN-induced thoracic aortopathy, and neither drug influenced disease initiation or progression. While we agree that single-cell RNA sequencing could offer deeper insights into downstream pathway mechanisms, we respectfully consider such analyses beyond the scope of the current study. We have revised the manuscript to emphasize the importance of future mechanistic investigations.

**Changes in the manuscript:**

Line: 295

Further studies are required to elucidate the precise mechanisms underlying BAPN-induced thoracic aortopathy. In particular, comprehensive histological analyses will be essential to identify the underlying cellular and molecular changes. In addition, single-cell RNA sequencing may provide deeper insights into downstream pathway mechanisms.

8) *Author did not included any data on PDE enzyme activity, cAMP/cGMP levels or any signaling related study which limits its focus why drugs were ineffective?*

**Authors’ reply:**

We appreciate these important suggestions. In response to the suggestions, we attempted to measure cAMP and cGMP abundance in aortic and lung tissues using ELISA kits (ADI-900-163, ADI-900-013, Enzo Biochem Inc.). However, we were unable to obtain rigorous and reproducible results, likely due to the extremely short half-lives of these second messengers and technical challenges associated with the difficulty of rapidly excising the aorta. To avoid potentially misleading interpretations, we have decided not to include these data in the manuscript. We have revised the Discussion section to emphasize the importance of assessing intracellular cAMP/cGMP signaling in future studies to better understand the pharmacological effects of PDE inhibitors in this model.

**Changes in the manuscript:**

Line: 319

Also, frequent plasma sampling and assessment of downstream signaling pathways, such as cyclic adenosine or guanosine monophosphates, are important to validate effective cilostazol and sildenafil administrations.

9) *The author states in the discussion section "These results indicate that BAPN-induced thoracic aortopathy is mediated by mechanisms distinct from other aortopathy models" by reinforcing this point with evidence or by emphasizing the ECM-dominant and non-inflammatory BAPN pathology that limits efficacy.*

**Authors’ reply:**

We have revised the manuscript to better highlight the distinctions between the BAPN-induced thoracic aortopathy model and other aortopathy models.

**Changes in the manuscript:**

Line: 293

These results indicate that BAPN-induced thoracic aortopathy is mediated by mechanisms primarily driven by extracellular matrix disruption, distinguishing it from other aortopathy models.

10) *This study lacks histological evaluation, my suggestion is to include immunostaining data which would deepen the mechanistic insights.*

**Authors’ reply:**

Thank you for the constructive suggestion. This study was designed primarily to investigate the impact of cilostazol and sildenafil on thoracic aortopathy. We agree with the reviewer on the importance of histological evaluation for deeper mechanistic insights if phenotypical changes are observed. Indeed, our previously publication that described the use of BAPN provided extensive histological characterization.(PMID: 38779856) However, our results did not demonstrate significant effect of either cilostazol or sildenafil on BAPN-induced thoracic aortopathy. We have revised the Discussion section to emphasize the need for future studies focused on mechanistic investigation, including detailed histological analyses.

**Changes in the manuscript:**
Line: 295

Further studies are required to elucidate the precise mechanisms underlying BAPN-induced thoracic aortopathy. In particular, comprehensive histological analyses will be essential to identify the underlying cellular and molecular changes. In addition, single-cell RNA sequencing may provide deeper insights into downstream pathway mechanisms.

11) *The author should explain the novelty of this study in relation to other published studies as the present condition of the study only reflects the characterization of two drugs efficacy in thoracic region otherwise author should provide mechanistictic explanation for their negative findings.*

**Authors’ reply:**

The novelty of our study is in evaluating the effects of cilostazol and sildenafil on thoracic aortopathy, that includes both aneurysm and dissection. While prior studies have reported opposing effects of these drugs in abdominal aortic aneurysm models, to our knowledge, no study has specifically investigated their roles in thoracic aortopathy. This is important because the thoracic aortic region and the abdominal aortic region exhibit distinct pathophysiological mechanisms. Therefore, the negative findings in our study are important in demonstrating that these agents do not exert similar influences in thoracic pathology. We agree with the reviewer that further mechanistic studies are needed. We have revised the Discussion section to emphasize the need for future investigation to elucidate mechanisms of these negative results.

**Changes in the manuscript:**
Line: 295

Further studies are required to elucidate the precise mechanisms underlying BAPN-induced thoracic aortopathy. In particular, comprehensive histological analyses will be essential to identify the underlying cellular and molecular changes. In addition, single-cell RNA sequencing may provide deeper insights into downstream pathway mechanisms.

12) *In the abstract section line 29 author started with the describing about the disease but why “Phosphodiesterases (PDEs) regulate intracellular cyclic nucleotide concentrations” suddenly came please explain.*

**Authors’ reply:**

The abstract has been revised for a more logical transition.

**Changes in the manuscript:**

Line: 29

Cilostazol, a phosphodiesterase (PDE) 3 inhibitor, and sildenafil, a PDE5 inhibitor, have been used clinically for peripheral arterial disease and erectile dysfunction or pulmonary hypertension, respectively. Recent studies report their effects on abdominal aortic aneurysm formation.

13) *In the Introduction section line 46 “A key pathological feature of thoracic aortopathy and changes” Please check the grammer.*

**Authors’ reply:**

The grammar error has been corrected.

**Changes in the manuscript:**

Line: 46

A key pathological feature of thoracic aortopathy is extracellular matrix (ECM) remodeling, including elastic fiber fragmentation and excessive collagen fiber deposition.

14) *In line 53 author mentioned “This mouse model” which mouse model author wants to mention, please include that.*

**Authors’ reply:**

The manuscript has been revised to clarify the mouse model.

**Changes in the manuscript:**

Line: 53

Therefore, BAPN administration may be an approach to investigate mechanisms and therapeutic potential of thoracic aortopathy.
